# On the Use of PLA-PHB Blends for Sustainable Food Packaging Applications

**DOI:** 10.3390/ma10091008

**Published:** 2017-08-29

**Authors:** Marina Patricia Arrieta, María Dolores Samper, Miguel Aldas, Juan López

**Affiliations:** 1 Institute of Polymer Science and Technology (ICTP-CSIC), Juan de la Cierva 3, 28006 Madrid, Spain; 2 Instituto de Tecnología de Materiales, Universitat Politècnica de València, 03801 Alcoy-Alicante, Spain; masammad@upvnet.upv.es (M.D.S.); miguel.aldas@epn.edu.ec (M.A.); jlopez@itm.upv.es (J.L.); 3 Departamento de Ciencia de Alimentos y Biotecnología, Facultad de Ingeniería Química y Agroindustria, Escuela Politécnica Nacional, Quito 170517, Ecuador

**Keywords:** food packaging, biopolymers, biodegradable, poly(lactic acid), poly(hydroxybutyrate), blends

## Abstract

Poly(lactic acid) (PLA) is the most used biopolymer for food packaging applications. Several strategies have been made to improve PLA properties for extending its applications in the packaging field. Melt blending approaches are gaining considerable interest since they are easy, cost-effective and readily available processing technologies at the industrial level. With a similar melting temperature and high crystallinity, poly(hydroxybutyrate) (PHB) represents a good candidate to blend with PLA. The ability of PHB to act as a nucleating agent for PLA improves its mechanical resistance and barrier performance. With the dual objective to improve PLAPHB processing performance and to obtain stretchable materials, plasticizers are frequently added. Current trends to enhance PLA-PHB miscibility are focused on the development of composite and nanocomposites. PLA-PHB blends are also interesting for the controlled release of active compounds in the development of active packaging systems. This review explains the most relevant processing aspects of PLA-PHB based blends such as the influence of polymers molecular weight, the PLA-PHB composition as well as the thermal stability. It also summarizes the recent developments in PLA-PHB formulations with an emphasis on their performance with interest in the sustainable food packaging field. PLA-PHB blends shows highly promising perspectives for the replacement of traditional petrochemical based polymers currently used for food packaging.

## 1. Introduction

Industrialization, urbanization, economic development and market globalization have led to worldwide changes in lifestyle and nutritional habits [1,2]. In fact, nowadays, most the food consumed is sold packaged not only to contain the food, but also to protect it during the whole production chain; that is, from the production to the place of sale or consumption. Therefore, the demand for safe, minimally processed, “fresh” food products has generated the need in the food industry to develop new packaging concepts and, nowadays, the major challenges for the food-packaging industry is to develop novel packaging systems for maintaining both the safety and quality of packaged foods [3].

**Figure 1:**
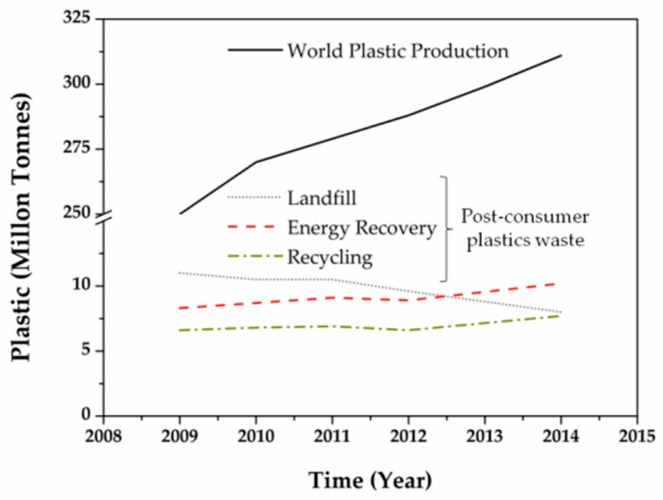
Progress of world plastic production and annual average of plastics post-consumer waste generation (data obtained from Plastics Europe [10]).

In this way, food packaging fulfill very important functions for the preservation of food products since they are required to protect foodstuff not only from external agents preventing physical, chemical and/or microbiological contamination, as well as from possible adulteration, but also avoiding the loss of food quality [4,5,6]. Food packaging also fulfill the function of providing information that is important for the consumer, as they also report the nutritional information, preferential date of consumption and how to conserve the foods that they contain. They may also contain information on the material from which the packaging is made, its recyclability and where it should be thrown away once it has reached its useful life. In this sense, plastics are the most demanding materials for food packaging applications due to practical and economical reasons including their low cost, lightness, easy of processing and easy to handling in integrated production lines in addition to their higher resistance than other materials such as ceramics, glass or cardboard [7,8]. In fact, high amount of the polymer production are demanded by the packaging sector. The final plastic packaging materials are predominately (between 70% and 99%) constituted of polymers (macromolecules composed of many repeated subunits) containing always various amounts of additives (plasticizers, antioxidants, pigments, antistatic, fillers and many other compounds) that are essential to provide the expected functionality to the final plastic product) [9]. Considering that the world plastic production has reached more than 250 million tons per year during the last years (thermoplastics and polyurethanes, Figure 1), joined with the fact that packaging products are commonly short term applications, they ultimately represent a big source of plastic wastes [10]. Therefore, the waste management strategies are focusing their attention on the material and energy recovery approaches. Although some fractions of the plastic waste can be recycled, most of packaging residues, and particularly those coming from the food packaging field, are disposed in landfills every year (Figure 1) due to both technical and economical reasons [11,12] creating an enormous amount of plastic waste, without energy or material recovery. Therefore, the use of non-renewable and non-biodegradable polymers for food packaging should be considered as potentially hazardous to the environment caused by both, the consumption of non-renewable petrochemical sources for their production as well as the accumulation of high amounts of plastic wastes after their useful life. These facts have led to an increasing concern on the use of more sustainable polymers for food packaging purposes known as biopolymers, including biobased and biodegradable polymers [12]. This strategy is aligned with the worldwide tend to develop a more sustainable economy which contributes to the growing up of the global production of bioplastics.

In this regard, the production and use of bio-based and biodegradable polymers, known as biopolymers, is currently increasing in the food packaging sector to reduce the consumption of non-renewable resources and prevent the accumulation of plastics waste, respectively. Biobased polymers are those polymers obtained from renewable sources, while biodegradable polymers are those which are able to degraded in simple molecules (water and carbon dioxide) under environmental conditions by the action of micro-organisms providing composting as a simple and sustainable disposal option [7,13,14]. Nowadays, many traditional polymers used in the packaging sector can be synthesized from renewable resources such as the case of the high density biopolyethylene (Bio-HDPE), derived from the bio-ethanol which is obtained from sugarcane, but with similar properties to those of conventional petroleum based HDPE [15]. However, for short-term food packaging applications, those plastics that combine the renewable origin and also biodegradable character are preferred to fill better the sustainable requirements to build a more sustainable circular economy.

## 2. Poly(lactic acid)

Among others, poly(lactic acid) (PLA) is the most used biobased and biodegradable polymer in the food packaging industry. PLA is chemically synthesized starting from simple sugars obtained from biomass and fermented to lactic acid. To produce PLA the most common route is the ring opening polymerization by condensation of lactide with metal catalyst, typically tin octoate, with the elimination of a water molecule at high temperature but less than 200 °C [9]. PLA is currently processed at industrial level with the same processing technology used for traditional petroleum-based thermoplastics. It is already commercialized mainly for single use disposal packaging applications such as bottles, cold drink cups, thermoformed trays and lids containers, blister packages, overwrap as well as flexible films [13,16,17]. Although PLA is nowadays economically competitive and it also possess many advantages for packaging purposes (i.e., ease of processing, superior transparency and environmentally benign characteristics) [13], it also shows some disadvantages such as the sensitiveness to thermal degradation, and poor barrier and mechanical performance, which hinder its industrial exploitation [13,14,16,17,18,19].

Considerable academic and industrial efforts have been focused on improving PLA performance to found more PLA commercial application in the sustainable packaging field, such as the copolymerization with other biopolymers [20,21] as well as by blending approaches [22,23,24,25,26,27]. From an industrial point of view, the modification of PLA by melt blending is particularly interesting because it is a moderately simple, cost effective and readily available processing technology at industrial level that allows obtaining simple packaging formulations with desired performance by varying the blend composition [28]. In fact, the compatibility of the components in the blend affects the physical properties of the final material such as glass transition temperature (*T_g_*), melting temperature (*T_m_*), degree of crystallinity (*X_c_*) and morphology. Accordingly, these properties determine the performance of the final material, i.e., processability, rigidity, impact and tensile strength, barrier properties and degradation behavior [29]. It is known that the increase of PLA crystallinity could improve its performance for food packaging applications, mainly because its direct impact on gas permeation properties [22]. In this sense, melt blending PLA matrix with another highly crystalline biopolymeric matrix with similar melting temperature has been considered as an easy way to increase PLA crystallinity and regulate its physical properties [27,28,30].

## 3. Poly(hydroxybutyrate)

The family of poly(hydroxyalcanoate)s (PHAs) has gain considerable attention in the food packaging industry [22,31,32,33,34]. PHAs are polyesters biologically synthesized by controlled bacterial fermentation for a wide variety of microorganisms (at least 75 different genera), but they have most commonly been studied on microorganisms such as Gram-negative bacteria (i.e., those belonging to the genera *Alcaligenes*, *Azobacter*, *Bacillus* and *Pseudomonas* [35]) as well as Gram-positive bacteria (i.e., those belonging to the genera *Rhodococcus*, *Nocardia* and *Streptomyces* [36]). PHA polymers are synthesized and accumulated by the bacterial cell in response to nutrient limitation as an intracellular food and energy reserve. In fact, under limited macro-elements (such as phosphorus, nitrogen, trace elements or oxygen) and in the presence of an abundant source of carbon (e.g., glucose or sucrose) or lipids (e.g., vegetable oil or glycerine) [9], bacteria can accumulate up to 60–80% of their weight in PHA to prevent starvation if an essential element becomes unavailable [36,37,38]. Among others, *Alcaligenes eutrophus* is one of the most commonly used organism for the production of PHA since it is easy to grow, it can accumulates up to 80% of dry cell weight in PHA in a simple medium and also its physiology as well as its biochemistry that lead to PHA synthesis are well understood [35]. PHAs also degrade by different bacteria, fungi, and algae in various environments. Regarding their properties, PHAs are isotactic semi-crystalline high molecular weight thermoplastic polymers [39]. Among PHAs, poly(hydroxybutyrate) (PHB) is the most simple and common representative of PHA [40], presents high crystallinity providing good gas barrier performance and thus widely studied for food packaging applications [41,42].

For plastic processing industry the main drawback of PHB is the very low resistance to thermal degradation. It presents the melting temperature (around 170–180 °C) close to the degradation temperature (at about 270 °C) [42,43]. For the food packaging industry the use of PHB has been limited mainly because of its high cost and brittleness with the consequent low strain at break [44]. Nevertheless, it is expected that the PHA production will almost quadruple by 2021 with regard to 2016, as a result of a ramp-up of capacities in Asia and the USA as well as the startup of a PHA plant in Europe (Bio-on, Italy) [45] and, thus, it seems that the costs of PHAs will decrease. To reduce PHA high crystallinity and improve its mechanical performance, blending with other polymers is considered to be an easy and cost-effective way [31,46,47].

## 4. PLA-PHB Based Polymer Blends

### 4.1. Miscibility and Processing Aspects of PLA-PHB Polymers Blends

The poor processability and formability of PHB is maybe the foremost drawback that limits its industrial uses and blending it with PLA represents an alternative way to introduce it in the market at the same time as PLA properties are improved. The effective blending of two polymeric matrices requires high affinity between them and it can be theoretically predicted by calculating the solubility parameters (δ) of each component of the blend since two substances with similar δ should be mutually theoretical soluble. The solubility parameter of PLA is between 19.5 MPa^1/2^ and 20.5 MPa^1/2^ [13], while that of PHB is between 18.5 MPa^1/2^ and 20.1 MPa^1/2^ [48]. Since the differences of the solubility parameter values are relatively low, good miscibility between both biopolymers should be expected [23]. However, the actual miscibility between PLA and PHB is also dependent on the processing temperature, the proportion of each polymer in the final blend as well as their molecular weight [22].

PLA-PHB blends have been extensively studied during the last years [40,49,50,51,52,53]. Blümm and Owen studied low-molecular-weight PLA (*Mn* = 1759) blended with high molecular weight PHB (*Mn* = 222,000, *Mw* = 794,000 g·mol^−1^) and they found that the PLA-PHB blends were miscible in the melt over the whole composition range, whereas a blend of high-molecular-weight PLLA (*Mn* = 159,400) with PHB showed biphasic separation [49]. It seems that PLA shows partial miscibility with low molecular weight PHB, particularly when the PHB content is about 25% [46]. Ohkoshi et al. studied PLA (*Mw* = 778,000 g·mol^−1^) blended with different molecular weights PHB (*Mw* = 9400 g·mol^−1^; 21,000 g·mol^−1^ and 140,000 g·mol^−1^) prepared by solvent casting technique using chloroform as solvent and further melt processed by compression molding at 200 °C, while samples were finally isothermally crystallized. They observed that the melt-compressed samples of PLA with low molecular weight PHB, *Mw* = 9400 g·mol^−1^, were miscible in the melt within the PHB content up to 50 wt % since the addition of PHB facilitated the crystallization of PLA, as revealed the study conducted by wide angle X-ray diffraction (WAXD) and DSC [54]. Similarly, Hu et al. studied PLA-PHB blends at different mass ratios (100:0, 80:20, 60:40, 40:60, 0:100) and their results indicated that PLA shows limited miscibility with low molecular weight PHB (*Mw* = 5000 g·mol^−1^) when the PHB content was below 25%. In fact, two crystals corresponding to the crystallization of both polymers were observed for the PLA-PHB 80:20 and PLA-PHB 70:30 blends. Whereas, no miscibility has been found between PLA and high molecular weight PHB (*Mw* = 650,000 g·mol^−1^) over the whole composition range [46]. Ni et al. blended PLA (*Mn* = 110,000 and *Mw* = 253,000 g mol^−1^) with oligomers of 3-hydroxybutyrate (OHB) with different molecular weights (*Mn* between 4000 and 83,000), obtained from thermally degraded PHB, to increase the miscibility between both polymers. They observed that OHB high-molecular weight (*Mn* = 4000) was able to enhanced PLA crystallization due to the formation of suitable size of spherulitic crystals which acted as effective nucleation agents for PLA. Meanwhile, the crystallization rate of PLA remained unchanged when it was blended with high-molecular weight OHB (*Mn* = 83,000) [51]. Additionally, when OHB was introduced in amounts lower than 40 wt %, it enhanced the PLA crystallization showing phase separation at higher loadings [51].

The processing temperature plays a decisive role on the PLA-PHB miscibility. In this context, PLA-PHB blends have been prepared by solvent casting technique at room temperature and it was observed that PLA (*Mn* = 43,000) and PHB (*Mv* = 300,000) were immiscible over the range of compositions studied (100:0, 80:20, 60:40, 40:60, and 0:100). Meanwhile, the same blend samples were then thermally treated by heating them up to 200 °C and these melt-blended samples showed greater miscibility, evidenced by a lower melting temperature of PHB as well as a lower *T_g_* for PLA, since the *T_g_* of neat PHB (around 0 °C) is lower than that of PLA (around 60 °C) [55]. This better miscibility has been ascribed to the fact that transesterification reactions take place between PLA and PHB chains during heating at 200 °C and thus it seems that PLA-PHB block copolymers can be produced in situ, compatibilizing both components in the polymeric blend [56]. Zhang and Thomas studied different formulations of PLA (*Mw* = 224,000 g·mol^−1^) and PHB (*Mw* = 283,000 g·mol^−1^) in 100:0, 75:25, 50:50, 25:75 and 0:100 proportions. In the case of PLA-PHB blend in 75:25 proportion they found well dispersed small spherulites of PHB in the amorphous PLA matrix. Whereas, for the PLA-PHB blends in 50:50 and 25:75 proportion crystalline PHB acted as the continuous phase [40]. On the other hand, PLA has been also blended with amorphous PHB: atactic poly[(R,S)-3-hydroxybutyrate] [57,58], a synthetic analog of bacterial PHB [58]. Poly[(R,S)-3-hydroxybutyrate] is an amorphous polymer chemically synthesized via ring-opening polymerization of racemic β-butyrolactone [57]. However, blends based on PLA with amorphous (R,S)-PHB resulted immiscible showing two glass transition temperatures, one between 0 °C and 2 °C and the second one around 60 °C, forming phase-separated polymeric blends [57,58].

The main conversion methods for PLA and PHB based materials are based on melt processing approaches (extrusion, injection molding, thermoforming, film forming, etc.) [31,59]. Prior to processing, both polymers should be dried for at least 4 h to prevent excessive hydrolysis (molecular weight drop), which can then compromise the performance of the PLA-PHB based materials on service. Amorphous PLA should be dried below the *T_g_*, that is between 43 °C and 55 °C, while semi-crystalline PLA drying temperature is at 80 °C [17]. Industrial grades of semi-crystalline PLA present the melting temperature in the range of 130–180 °C [59,60]. Since PHB presents the melting temperature around 170–180 °C, the processing temperature of PLA-PHB blends should be at least 180–190 °C. Melt processing approach involves heating PLA-PHB blends over their melting points, modeling the polymeric blend to the desired forms and, to finish, cooling to stabilize its dimensions.

PLA and PHB interfacial compatibilization can be improved by melt reactive extrusion. For instance, Jandas et al. used maleic anhydride (MA) as reactive compatibilizer for PLA and PHB matrices to increase not only their miscibility, but also to increase the flexibility of the final blends. The reactive extrusion as compatibilization technique follows the grafting mechanism of MA on the α-carbon atom of the carbonyl group of PLA and PHB [61]. Their compatibility has been also improved by the formation of branching/partial crosslinking structure at their interfaces by means of the presence of dicumyl peroxide [62].

Another interesting approach for processing PLA-PHB blends without temperature is by means of the electrospinning technique [47,63,64]. Electrospinning technology allows the production of long and continuous polymeric fibers starting from a polymeric solution and at room temperature, that allows obtaining electrospun fibers with large surface areas, small inter-fibrous pore size with high porosity in the form of non woven mat [65]. Recently, Arrieta et al. processed PLA-PHB solutions at different mass ratios (100:0, 75:25, 50:50, 25:75, and 0:100) and the results indicated that the best electrospun mat formulations was PLA-PHB at 75:25 proportion due to a number of structural interactions between PLA and PHB, as revealed by Fourier transform infrared spectroscopy (FTIR) and Raman assays [47].

#### 4.1.1. Plasticization of PLA-PHB Blends

Both PLA and PHB are brittle and, thus, the PLA-PHB blends have poor processing properties as well as the final materials result in a high modulus and strength, but also high brittle with a lack in toughness. These drawbacks are especially disadvantageous in the film extrusion industry [29]. Therefore, several strategies have been proposed to improve their processability including blending them with a third component, such as the addition of copolymers derived from hydroxyl alkanoic acids or plasticizers [5,22,23,66,67] as well as through the development of composites [64,68] and/or nanocomposites [43,55,69]. From an industrial point of view, blending is much more easy and cost-effective than copolymerization and, as a result, the more frequently used method in the industrial sector [29].

The use of plasticizers, which are frequently added at industrial level to get flexible materials for film manufacturing, also favors the processing of polymeric matrices due to the increased polymer chain mobility. In this sense, PLA-PHB plasticization has been proven to be an effective way to increase the blend flexibility as well as to improve the compatibility between PLA and PHB biopolymers [5,22,23,66,70,71]. It is known that compatibility between polymer blends is a major issue for effective plasticization [23,72], and thus, the use of common plasticizers used for PLA as well as for PHB plasticization is the most used strategy to plasticize the PLA-PHB blends. Several plasticizers have been used for PLA mainly at concentrations between 10 wt % and 30 wt % for film applications, such as: glycerol [73], oligomeric lactic acid (OLA) [73,74]; poly(adipates) [75]; poly(ethylene glycol) (PEG) [23,76]; citrate esters, including tributyl citrate (TbC) [29,73], triethyl citrate (TEC) [77] and acetyl tri-n-butyl citrate (ATBC) [23,72,76,78] and low molecular weight additives have also been investigated as potential plasticizers for PLA such as aroma compounds including D-limonene [53,79,80,81], carvacrol, thymol [82,83], among others [84]. PHB plasticization has been performed with glycerol [85,86], glycerol triacetate [86], PEG [85], ATBC [87,88], tri(ethylene glycol) bis(2-ethyl exanoate) (TEGB) [85], pentaerythritol (penta) [85], among others. Along with all kind of plasticizers, nowadays there is an industrial trend to change traditional plasticizers for natural plasticizers due to the migration phenomenon, which could result in potential human health and environmental hazards [89,90].

Several authors have added plasticizers to increase the PLA-PHB compatibility and to facilitate their processing as well as their application as flexible films. For instance, Abdelwahab et al. blended PLA (*Mw* = 52,000 g·mol^−1^) with PHB (*Mw* = 425,000 g·mol^−1^) in 75:25 proportion and the PLA-PHB blend exhibited two *T_g_* values suggesting some kind of immiscibility. The PLA-PHB blend was further plasticized with a polyester plasticizer (Lapol 108, *Mw* = 80,000 g·mol^−1^) derived from more than 50% renewable resources. Lapol 108 essentially decreased the *T_g_* of PLA from 62 °C in PLA-PHB blend to around 58 °C using 7 wt % of Lapol plasticizer, while the elongation at break increase from 7% to 15% [66]. Arrieta et al. blended PLA (*Mn* = 217,000 g·mol^−1^) with PHB (*Mw* = 416,000 g·mol^−1^) in 75:25 proportion and further plasticized the PLA-PHB blend with 15 wt % of different additives; the aroma compound D-limonene (*Mw* = 136 g·mol^−1^) [22,53,91] as well as with two plasticizers: PEG (*Mn* = 300 g·mol^−1^) [23] and ATBC (*Mw* = 402 g·mol^−1^) [5,22,23]. In all cases the plasticization effect improved the processability between both biopolymeric matrices by melt blending [5,22,23,92,93] and also by electrospinning approach [47,63,64]. D-limonene was able to reduce the *T_g_* of PLA from 58 °C in PLA-PHB blend to around 39 °C in PLA-PHB-LIM and increased the elongation at break from 2% to 8% [53]. In this context, plasticizers PEG and ATBC were more effective reducing the *T_g_* reaching 25 °C and 31 °C, respectively. The elongation at break of PLA-PHB-PEG resulted in 6%, while ATBC showed significant improvements since PLA-PHB-ATBC achieved 180% in elongation at break [23]. The higher plasticization effect has been also related with their high similarity in solubility parameters among PLA (δ = 19.5–20.5 MPa^1/2^ [13]), PHB (δ = 18.5–20.1 MPa^1/2^ [48]) and ATBC (δ = 20.2 MPa^1/2^ [23]) plasticizer in comparison with PEG (δ = 16.7 MPa^1/2^ [23]) and D-Limonene (δ = 14.3 MPa^1/2^ [91]), which showed lower solubility parameters than those of PLA and PHB, but still in the same order of magnitude.

At the high temperature required for polymer processing, some plasticizer evaporation could take place because of they are in general less thermally stable than polymeric matrices. For this reason, the use of aroma compounds as additives for PLA-PHB blends presents an important inconvenience related with the fact that they have low molecular weights and, in general, are highly volatile for melt/extrusion purposes. The remaining amount of D-limonene in ternary PLA-PHB-Limonene blends after processing has been calculated by pyrolysis coupled with gas chromatography and mass spectrometry (Py-GC/MS), and it was observed that around 30% of D-limonene was lost during processing [53]. Nevertheless, the loss of D-limonene was less in PLA-PHB blends plasticized with 15 wt % of D-limonene (PLA-PHB-LIM) than in PLA plasticized with 15 wt % of D-limonene (PLA-LIM), in which above of 40 wt % of D-limonene was lost during processing [79]. Armentano et al. blended PLA (*Mn* = 14,000 g·mol^−1^) with PHB in 85:15 proportion and then incorporate 10 wt % of carvacrol and further plasticize the system with OLA (*Mn* = 957 g·mol^−1^). The remaining amount of carvacrol in PLA-PHB-Carvacrol and PLA-PHB-OLA-Carvacrol films after processing was determined by High Pressure Liquid Chromatography (HPLC) and they found that approximately 25% of the carvacrol was lost during processing in both formulations [70]. The *T_g_* of PLA-PHB (55 °C) was reduced to 47 °C in PLA-PHB-Carvacrol and to 36 °C in PLA-PHB-OLA-Carvacrol, highlighting the positive effect of OLA as plasticizer [70]. OLA, with a similar chemical structure of PLA, has a solubility parameter of 17.7 MPa^1/2^ [94], while that of carvacrol is lower (δ = 15.1 MPa^1/2^) [95]. The elongation at break of PLA-PHB of 100% was not significant improved with the addition of carvacrol (ε_%_ PLA-PHB-Carvacrol = 105%), but it was significant improved with the addition of both, OLA and carvacrol, ε_%_ PLA-PHB-OLA-Carvacrol = 150% [70].

#### 4.1.2. PLA-PHB Based Masterbatch, Composites and Nanocomposites

The use of a preformed masterbatch is a widely used approach in the polymer-processing industry that leads to more homogeneous blend and enhances the performance of the final material [7,96,97]. In the case of PLA-PHB blends since both polymers present similar melting temperatures they can be well blended in the melt state [43]. The preparation of PLA-PHB masterbatch involves the pre-mixture of both polymeric matrices and further processing into the final material by adding different additives, including the development of plasticized PLA-PHB materials, PLA-PHB based composites or nanocomposites [5,28,43]. In this sense, the development of composites and nanocomposites is also a widely use approach that is growing up in the packaging industrial sector since the addition of micro and/or nano reinforcements leads to an improvement in the thermomechanical performance of biobased and biodegradable polymeric matrices. In the case of PLA-PHB based composites and nanocomposites, to guarantee the packaging’s green nature, natural fillers are preferred [22].

In plasticized systems, the masterbatch approach facilitates the homogeneous distribution of plasticizer between PLA and PHB polymeric chains [5]. Meanwhile, in PLA-PHB based composites and nanocomposites, the masterbatch-method favors the processability of the filler by reaching a more homogeneous distribution of fillers and/or nanofillers into the polymeric PLA-PHB matrices which further allow fillers to promote a higher nucleation effect [28,43].

### 4.2. PLA-PHB Polymers Blends Properties

#### 4.2.1. Thermal Properties and Crystallization Behavior of PLA-PHB Polymers Blends

##### Thermal Stability

**Figure 2:**
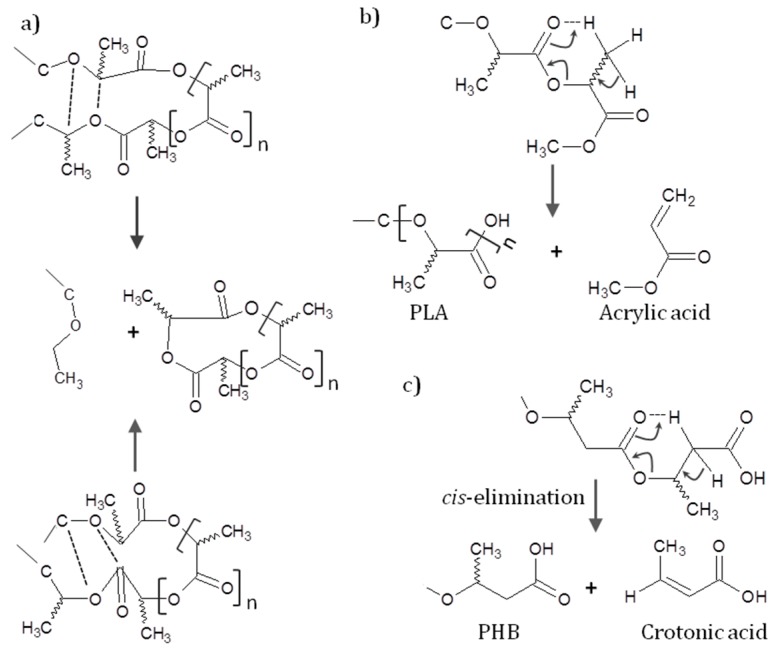
Non-radical thermal degradation of PLA: (**a**) trans-esterification; (**b**) *cis*-elimination (adapted from Kopinke et al. [101]); and (**c**) non-radical thermal degradation of PHB *cis*-elimination (adapted from Aoyagi et al. [102]).

As it was already commented, the main drawback of PLA-PHB based formulations for melt processing is the low thermal stability of PHB. The onset degradation temperature of PHB is between 245 and 260 °C [23,98,99]. The processing temperature of PLA-PHB is determined by the melting temperature of PHB which is relatively high around 170–180 °C. Consequently, the processing temperature should be at least 180–190 °C and, considering that its maximum degradation temperature is at about 270 °C, PHB shows a small processing window [35,43,46,100]. In the case of PLA, its thermal decomposition at temperatures above 200 °C involves mainly two thermal degradation mechanisms: (i) intra- and intermolecular trans-esterification that leads to the formation of lactide and cyclic oligomers (Figure 2a); and (ii) *cis*-elimination (Figure 2b) that leads to the formation of acrylic acid and acyclic oligomers [101]. Meanwhile, the thermal degradation of PHB mainly involves non-radical random chain scission reaction (*cis*-elimination), which results in a rapid decrease in its molecular weight (Figure 2c) [87,102]. On the other hand, when PHB possesses the terminal groups in the form of carboxylate salt, the carboxylate end groups induce degradation of PHB at moderate temperatures and the degradation via intermolecular α-deprotonation by carboxylate has been suggested as PHB decomposition pathway at temperatures above 120 °C [103].

To simulate the industrial processing conditions PLA-PHB based materials in 75:25 proportion have been studied by isothermal thermogravimetric analysis (TGA) at the processing temperature. It was observed that plasticized PLA-PHB samples with ATBC and PEG showed improved thermal stability than PLA-PHB under isothermal TGA analysis conducted at 180 °C for times lower than 6 min, which was the processing whole time used for melt blending and film forming process, losing less than 1% of the initial mass [23]. Similarly, the thermal stability of PLA-PHB masterbatch and the corresponding nanocomposites reinforced with cellulose nanocrystals (CNC) and functionalized cellulose nanocellulose by means of the use of the surfactant acid phosphate ester of ethoxylated nonylphenol (CNC-s) were also studied under isothermal TGA mode at the highest extrusion temperature of 200 °C. After the actual processing time of 7 min at 200 °C the PLA-PHB-CNC nanocomposite lost approximately 5% of the initial mass resulting in lesser thermally stability than PLA-PHB masterbatch, while better dispersed CNC-s leads to a comparable mass loss than PLA-PHB masterbatch (mass loss lower than 3%) [43].

The addition of PHB decreases the thermal stability of PLA, as reveals dynamic TGA analysis, where PLA-PHB blends show the thermal degradation in two-steps process, with the first degradation step related to the PHB decomposition and the second one to the PLA degradation [23]. Nevertheless, it should be mentioned that the degradation temperatures of PHB component in PLA-PHB blends are higher than that of the neat PHB, while reduced PLA thermal stability is observed on the second degradation stage with a minor decrement [23,104]. The fact that both degradation curves, that of PHB and that of PLA, become closer has been attributed to the PLA and PHB good compatibility with PLA serving as a shielding barrier to delay the thermal degradation process of the PHB component in the blend [104].

##### Crystallization Behavior

Because PLA-PHB blends belong to the family of semi-crystalline/semi-crystalline polymer blends, the crystallization behavior of each component in the blend is dependent on their miscibility, physical properties and crystallization conditions. For example, the crystallization of one component affects the morphology, crystallization, and mechanical properties of the other [46]. PHB is mainly added to PLA matrices since it could enhance its crystallinity [51]. The increase in PLA crystallinity became interesting for food packaging applications in order to increase the barrier performance of PLA based materials. It is known that PLA can crystallize showing different polymorphisms known as the alpha (α), beta (β), and gamma (γ) crystals as well as the alpha prime (α′) which are mainly disorder α crystals [105]. From the melt, PLA crystallizes in the α form at temperatures higher than 120 °C [13], while the α' form appears when PLA sample crystallized below 110 °C [105].

The crystallization and melting behavior of PLA (*Mw* = 200,000 g·mol^−1^) blended with low molecular weight PHB (*Mw* = 5000 g·mol^−1^) were investigated by Hu et al. They observed a remarkable effect on the cold crystallization of PLA in the blends due to the addition of PHB. When PHB was added in at least 20–30 wt % two crystals appears, that correspond to the crystallization of PLA and PHB as revealed the infra-red (IR) analysis conducted during the heating process. Additionally, the disorder (α′) phase of PLA was produced in the nonisothermal crystallization process [46]. Since PLA and PHB are semi-crystalline polymers their FTIR spectral profiles are greatly affected by their corresponding physical states and crystalline structures [46]. In fact, due to their differences in the state of crystalline order, PHB is more crystalline than PLA, and the carbonyl bands differ significantly in FTIR [52]. The typical asymmetric stretching of the carbonyl group of PLA is between 1745 cm^−1^ and 1755 cm^−1^, and it is attributed to the amorphous carbonyl vibration [40,46,47]. The crystalline carbonyl stretching vibration of PHB is centered around 1720 cm^−1^, while near 1740 cm^−1^ appears a band related with the amorphous state of PHB [40]. Thus, the changes on the intensity ratio of carbonyl bands with the composition ratio of PLA-PHB in the blends are widely studied to better understand the miscibility between PLA and PHB. Zhang and Thomas studied PLA-PHB blends at different composition ratios (100:0, 75:25, 50:50, 25:75 and 0:100) and they observed that for the PLA-PHB 75:25 blend, the FTIR spectrum is different from the other blends which showed the carbonyl groups clearly individualized. PLA-PHB 75:25 blend the carbonyl band at 1745 cm^−1^ become broadened, while that of the crystalline PHB was shifted to lower wavelengths, suggesting positive interactions between PLA and PHB that has been ascribed to transesterification reactions [40].

**Figure 3:**
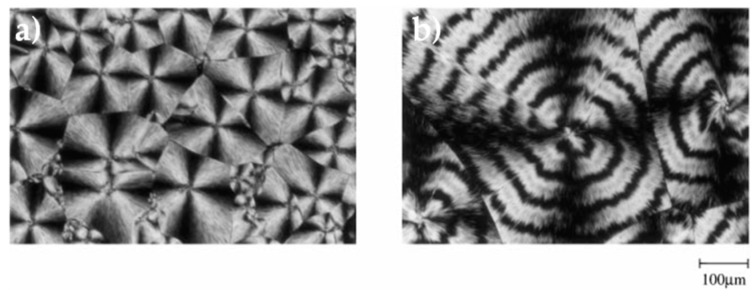
Optical micrographs of PLA spherulites in: (**a**) neat PLA; and (**b**) PLA-PHB (75:25) blend crystallized at 130 °C from the melt (200 °C). Reprinted with permission from [54].

Ohkoshi et al. isothermally crystallized PLA (*Mw* = 778,000) blended with PHB (*Mw* = 9400) in 75:25 proportion after melting at 200 °C and studied the crystallization by means of cross-polarized optical microscopy (Figure 3). In neat PLA sample the typical optical micrographs of the PLA spherulites are observed, while PLA-PHB sample crystallized at 130 °C from the melt showed that the PLA spherulites present the obvious banding morphology in which spherulite radius increased linearly with time, indicating that the PHB component is trapped into PLA spherulites [54]. Another interesting and related phenomenon observed for PLA-PHB blends containing 25 wt % of PHB was observed by polarized optical microscopy by Blümm and Owen (1995), who found that several minutes after the formation of PHB spherulites the birefringence suddenly changed from positive to negative, indicating that the crystallization of PLA within the interlamellar regions of the PHB spherulites had occurred [49].

It seems that the well interaction of both polymers melt blended in 75:25 proportion is due to some transesterification reactions between PLA and PHB chains take place during melting [40,56]. Actually, the highly ordered stereochemical structure of PHB crystallizes as small spherulites that are well dispersed in the amorphous PLA matrix and are able to act as nucleating agents for PLA [40], increasing its crystallinity [22,23,43,66] and, thus, improving the film final barrier performance [22,23,28]. As a result, melt blending PLA with 25 wt % of PHB have gained special attention in the plastic processing industry for the development of materials for food packaging applications.

Tri et al. (2013) developed PLA-PHB in 90:10 proportion based composites loaded with talc (from 0.5 to 5 phr) and in order to simulate typical industrial processing approaches, such as extrusion or injection molding, they further studied the obtained biocomposites by non-isothermal crystallization. They observed that although 10 wt % of PHB shows a nucleating effect for PLA matrix it seems to be not efficient enough to allow PLA full crystallization. The combined effect of PHB and talc promoted the cold crystallization of PLA matrix, while no significant differences were observed for higher talc content than 1 phr [55]. PLA-PHB blends has been also loaded with catechin (Cat) and further plasticized with ATBC. Catechin presence also promoted faster crystallization in PLA-PHB (PLA-PHB-Cat) and in plasticized PLA-PHB based materials (PLA-PHB-ATBC-Cat) and it was ascribed to hydrogen-bonding interactions between catechin hydroxyl groups and carbonyl groups of PLA, PHB and ATBC [93]. The hydrogen-bonding interactions, that are favoring the interaction among all the components in the blend, has been also observed in bionanocomposites based on PLA-PHB (PLA-PHB-CNC) blends and plasticized PLA-PHB blends reinforced with cellulose nanocrystals (PLA-PHB-ATBC-CNC). In fact, the combination of cellulose nanocrystals with the plasticizer in the PLA-PHB blends produced a synergic effect on the crystallization of PLA leading to higher cold crystallization temperature values and higher crystallinity degrees [5].

#### 4.2.2. Mechanical Performance of PLA-PHB Polymers Blends

Both PLA and PHB are fairly hard and brittle materials, and not very useful for many industrial applications. The mechanical performance of PLA is characteristic of glassy polymers with low deformation at break, while neat PHB has a low melt viscosity and it is a brittle polymer with a higher modulus [66]. The overall effects of the addition of high crystalline PHB on the PLA tensile properties are the increase in Young modulus (*E*) and tensile strength (*TS*) accompanied with a reduction of the elongation at break (ε*_B_*) [23,47,66,70]. Table 1 summarizes the mechanical properties analyzed by means of tensile test assays of several PLA-PHB based formulations.

PLA-PHB films showed Young modulus significant higher than neat PHB and neat PLA [22]. The tensile strength and the elongation at break of PLA-PHB blends decrease with PHB content [40]. For higher contents of PHB, in those blends in which it is the continuous phase both, tensile stress and elongation at break, result lower than those of neat PLA [40]. Nevertheless, the PLA-PHB 75:25 blend shows better mechanical performance than neat PLA confirming that the finely dispersed PHB crystals acts as a filler for PLA matrix [40]. In fact, PLA-PHB blends with a high fraction of PHB (up to 60 wt %) behave as brittle polymeric systems and break at very low elongations (<3%), while PLA-PHB blends with lower than 40 wt % of PHB can be stretched up like typical thermoplastic polymers [52]. Similarly, the impact resistance of PLA-PHB 75:25 results higher than that of homopolymers [107]. Thus, it seems that a synergic effect take place in PLA-PHB 75:25 blend since this blend formulation showed higher mechanical performance than neat PLA [40], while the PHB high rigidity is reduced [52].

Zhang et al. showed by dynamic mechanical (DMA) analysis that the softening of PLA can be reduced above 60 °C, by blending it with PHB. While the storage modulus of neat PLA is almost constant at temperatures below the *T_g_* and then drops at the *T_g_*, the storage modulus of PLA-PHB blends in 75:25 and 50:50 proportion at temperatures below the *T_g_* of PLA resulted higher than those of neat PLA and then increases, indicating the recrystallization of PLA. They also observed that these curves gradually decreased in storage modulus from around 30 °C, which is associated with the glass transition of PHB. However, for higher amounts of PHB than 25 wt % in the blend, the recrystallization of PLA was difficult to observe since the storage modulus shows a gradual but small decrease from about 60 °C [40]. Zhang et al. studied the fracture surface of PHB by SEM, and observed that neat PHB, which is a very brittle material, is drastically modified due to the second phase of PLA, concluding that the PLA-PHB blends are expected to have better fracture toughness [56].

Packaging materials intended for film applications require high flexibility and, thus, in film processing industry plasticizers are frequently added. The elongation at break increase from 7% in PLA-PHB (75:25) to 15% in PLA-PHB-Lapol7%, without significant reduction of Young Modulus and Tensile strength [66]. The incorporation of PEG and/or ATBC produced a reduction in 70% in *E* and 65% in *TS* when compared with neat PLA, since plasticizers induced ductile fracture. The addition of PEG did not show any clear improvement in ductile properties of PLA and PLA-PHB 75:25 blend. ATBC in 15 wt % produced much higher increase in ductility than most of plasticized PLA-PHB based materials at this proportion (i.e., PEG, Lim, OLA, etc.) reaching 180% of ε*_B_* for PLA-PHB-ATBC in (75:25):15 [23]. Higher amounts of plasticizers have also been added, for instance, PLA-PHB 85:15 has been tuned from rigid to ductile by adding OLA plasticizer from 15 wt % to 30 wt %. While 15 wt % of OLA content produced very poor ductile material, the addition of 30 wt % of OLA produced high flexible materials (>350% in ε*_B_*) [71]. Jandas et al. further increased the flexibility of PLA-PHB by compatibilizing them by grafting of maleic anhydride (MA) by reactive extrusion approach. The PLA-PHB 70:30 changed from a brittle material to a ductile material for PLA-PHB-MA blends as a function of MA addition (from 1 wt % to 9 wt %), reaching the optimum flexibility of more than 500% by grafting 7 wt % of MA (PLA-PHB-MA) [61].

The mechanical resistance of PLA-PHB materials has been improved by the development of composites and nanocomposites. The fillers, such as catechin, as well as nanofillers, such as nanocellulose, organically modified nanoclay (OMMT) and/or modified montmorillonite Cloisite 30B (C30B), produce a reinforcing effect into PLA-PHB matrices owing to the enhancement of the biopolymers’ interfacial adhesion in the blend, leading to more homogeneous material in the final blend composites and nanocomposites [28,61,93]. The foremost advantage of developing nanocomposites, instead of composites, is that, if good dispersion of the nanofiller is achieved, the overall mechanical performance of PLA-PHB can be improved. In this context, PLA-PHB blend matrix has been reinforced with cellulose nanocrystals (CNC) and functionalized CNC by means of a surfactant (CNC-s). Functionalized CNC-s were better dispersed than non-functionalized CNC as revealed TEM and AFM analysis and thus, ternary PLA-PHB-CNCs showed the better mechanical performance [28]. To obtain highest elongation at break a plasticizer was further added. The plasticized quaternary bionanocomposites loaded with modified CNC-s (PLA-PHB-CNCs-ATBC) achieved the highest elongation at break of about 150%, with respect of the non-functionalized CNC based nanocomposites [5], showing comparable values to those of commercial stretchable films used for food packaging. The well dispersion of the nanofiller into the polymeric matrix has been ascribed not only to the functionalization of CNC-s that avoid CNC agglomeration [43], but also to the presence of plasticizer that improves the dispersion of fillers in the polymeric matrix [5]. Compatibilized PLA-PHB blends using MA resulted in high flexible materials. The reduced stiffness has been compensated by developing nanocomposites reinforced with nanoclays OMMT and C30B. The composition with 3 wt % loading of both nanoclays showed the best balance for the mechanical performance required for packaging uses [61].

It should be mentioned that the performance of PLA and PHB as well as their blends could suffer changes over time due to the inherent biodegradability of both polymeric matrices [93]. In this context, tensile tests were performed after two years of processing PLA, PHB and PLA-PHB blends, and this aging study have showed significantly increase in ductility over the ageing time [107]. Concerning their application in short term applications intended for the food packaging field, the performance of the packaging material should be guaranteed at least during the food’s shelf life [108]. To corroborate their potential application as packaging material Arrieta et al. studied the nanomechanical and structural changes of PLA-PHB based blends after their useful life. With this purpose, PLA-PHB based composites loaded with catechin and further plasticized with ATBC were exposed during 10 days to a fatty food simulant. A drastic diminution of the nanomechanical performance was observed for PLA-Cat, PLA-PHB-Cat and PLA-ATBC-Cat active materials as a result of the release of catechin to the food simulant. Nevertheless, the quaternary formulation PLA-PHB-ATBC-CAT mainly maintained the mechanical properties of PLA-PHB-ATBC after the release of catechin to the food simulant [93].

#### 4.2.3. Optical and Barrier Properties of PLA-PHB Polymers Blends

##### Visual Appearance, UV Blocking and Colorimetric Aspects

The visual appearance of films is an important consumers requirement for materials intended to be used for food packaging applications [32,70,79,106]. In this sense, one of the most important aspects of packaging materials for consumer acceptance is their transparency [109]. While PLA is highly transparent and colorless, neat PHB presents amber tonality and lower transparency in the visible region of the spectra (400–700 nm). Haugaard et al. studied the color changes in PLA and PHB exposed to food simulant. The color changes in PLA as well as in PHB were almost comparable to that occurred in traditional plastics used in the packaging field such as HDPE [110], confirming the interest of PLA and PHB for food packaging applications. The amber tonality of PHB characterized by a positive values for both a* coordinate (green-red) and mainly for b* coordinate (blue-yellow) in the CIELab space, can be successfully reduced by blending with PLA (PLA-PHB 75:25) [22,23,28]. The addition of other additives to PLA-PHB blends such as plasticizers (PEG [23], ATBC [5,23], OLA [71]), essential oils (D-limonene [53] and carvacrol [71]) and nanoparticles (CNC [5,28]) maintained the transparency or even improved it by decreasing their yellowish trend, which can be measured by the decrease of the yellowness index with respect to PLA-PHB counterpart [22,23,28].

The development of transparent films with increased ultraviolet (UV) protection is another relevant issue in food packaging, since food packaging should also protect foodstuff from UV light. In this sense, another advantage of adding PHB into PLA matrix is that PHB acts as a better light barrier in the UV light region (250–400 nm) than PLA [32]. For instance, in PLA-PHB/nanocellulose based nanocomposites PHB as well as nanocellulose (CNC), particularly well dispersed functionalized CNC-s, produced somewhat blocking effect on the ultra violet C region (280–100 nm) [5,28], which is usually produced by artificial light sources [13].

#### 4.2.4. Wettability and Barrier Performance

##### Surface Wettability

Surface wettability is a relevant property for materials intended for food packaging since it will directly influence many other properties of polymers such as the permeability towards water vapor, selective adsorption, adhesion, printing, controlled release of molecules, the beginning of biodegradation process, etc. [7]. Static water contact angle (WCA) values lower than 65° are related with hydrophilic surfaces, while higher values than 65° are related with hydrophobic surfaces [111]. Although PLA and PHB are relatively hydrophobic polymers, with static water contact angle values higher than 65° [23,80,112,113], they still show higher wetting performance than traditional petroleum based polymers with higher static water contact angles around 100°, such as LDPE [114] and PP [115].

Several efforts have been put in increasing the hydrophobicity of PLA matrix in order to protect food products from the effects of moisture and humidity during storage. In this sense, PLA-PHB (75:25) blends showed a significant improvement in their hydrophobic character with reduced water affinity, PLA-PHB: 70°–90° [5,23,28]. Different behavior has been observed for plasticized PLA-PHB (75:25) matrices. For instance, the use of 15 wt % of ATBC and particularly 15 wt % of PEG resulted in a slight increase in the hydrophilic character of PLA-PHB surface: PLA-PHB-ATBC WCA = 67° ± 3° and PLA-PHB-PEG WCA = 57° ± 2° [23]. The increased hydrophilicity has been related with the fact that the plasticizing effect influences the diffusion process as a consequence of the increased polymer chain mobility [80]. Meanwhile, the addition of D-Limonene produced an increase in the surface hydrophobicity of PLA-PHB (PLA-PHB-Lim WCA = 74° ± 1°). These findings have been related with the more hydrophobic nature of D-Limonene [53].

The wettability performance of materials is not only strongly dependent on the surface chemical properties, but also on the topographical features of the material surfaces [80,116]. PLA-PHB (75:25) blend nanocomposites loaded with 5 wt % of hydrophilic nanoparticles, such as CNC, caused an increase in surface wettability (PLA-PHB-CNC WCA = 74° ± 3°) with respect of PLA-PHB blend (PLA-PHB WCA = 90° ± 2°), while the functionalization of CNC by means of a surfactant (CNC-s) leads to a not significantly change of the PLA-PHB surface wettability (PLA-PHB-CNCs WCA = 91° ± 2°). The synergic effects of the more crystalline PHB and functionalized CNC-s on the PLA produced an increase on surface hydrophobicity of the final nanocomposite (PLA-PHB-CNCs) [28] and this could be related with the establishment of hydrogen bond interactions between carbonil groups of PLA and PHB with hydroxyl groups of nanocellulose [5]. The further plasticization of PLA-PHB-nanocellulose based nanocomposites with 15 wt % of ATBC increased the surface hydrophilicity of PLA-PHB films showing WCA values between 68° and 70° [5].

It should be highlighted that in general all PLA-PHB based formulations showed WCA values higher than 65°, being materials acceptable for the intended end-use applications as food packaging materials.

##### Oxygen Permeability and Water Vapor Properties

In spite of the similarity of PLA and PHB molecular structures, their barrier performance of PLA and PHB is very different since they present real differences in molecular stereo-regularity, crystallinity degree and the glass transition temperatures [117]. The oxygen transmission process through a packaging material could be determined by calculating the oxygen permeability coefficients (OPC, kg·mm^−2^·s^−1^·Pa^−1^) which indicates the amount of oxygen that permeates per unit of area and time in a packaging material or by the oxygen transmission rate per film thickness (OTR*e, cm^3^·mm·m^−2^·day^−1^), thus taking into account the influence of material thickness in the permeation process [94,118]. PLA and PHB posses better oxygen barrier performances than that of LDPE (OTR*e: 160 cm^3^·mm·m^−2^·day^−1^ [94]), but lower than that of PET (OTR*e: 3 cm^3^·mm·m^−2^·day^−1^ [94]). The PHB oxygen as well as water vapor barrier performance is better than that of PLA (Table 2). In fact, the family of PHAs are highly hydrophobic, showing water vapor barrier properties in the range of other petroleum based conventional thermoplastics, such as PET (5.2 × 10^−15^) [119].

It is widely known that PLA, and particularly plasticized PLA, presents low oxygen barrier performance, being one of its most important drawbacks for food packaging purposes. Plasticizers can also increase the water permeability, reducing the water barrier performance of packaging materials due to the higher polymer chain mobility and increased free volume. Similarly, the WVP of plasticized PHB can result in an increase of one order of magnitude in comparison with WVP values of neat PHB, as reported D’Amico et al. for PHB plasticized with 20 wt % of tributyrin [106].

The strategy to blend PLA with more crystalline PHB results in a important improvement on the oxygen barrier properties, particularly in the case of plasticized system where the plasticizer increase the polymer chain mobility [23,79]. For instance, PLA and PLA-PHB blends has been plasticized with D-Limonene [22,53,79], PEG [23], OLA [67,70] and ATBC [5,23]. While no significant improvement on plasticized PLA oxygen barrier performance was observed for the PLA-PHB 75:25 plasticized with 15 wt % of PEG [23], the addition of 25 wt % of PHB induced a reduction in oxygen permeability of PLA plasticized with D-Limonene [53] and ATBC [5,23]. In the case of PLA-PHB systems plasticized with PEG, where the oxygen barrier performance remained unchanged in comparison with PLA-PEG, it seems that the PLA crystallization caused by physical aging induces plasticizer segregation towards the amorphous phase increasing the free volume around macromolecular chains in the polymer structure [76]. Meanwhile, a significant reduction in the OTR*e values of PLA-Lim and PLA-ATBC was observed in ternary PLA-PHB-Lim (up to 35%) [53] and PLA-PHB-ATBC (up to 55%) [23], attributed to the well interaction among all components in the blend. Similarly, Armentano et al. observed that the addition of 20 wt % of OLA plasticizer to PLA-PHB (85:15) blend produced a significant increase in the OTR*e values of PLA-PHB 85:15 (up to 70%), due to the increase in the free volume of the polymeric matrix. Nevertheless, higher amounts of OLA of 30 wt % showed no significant differences in OTR*e values while slightly reduced the WVP of PLA-PHB (85:15) blend. Authors attributed this result to the higher crystallinity developed in this ternary system revealed by X-ray diffraction and DSC analysis which ultimately induces major tortuosity for the oxygen and water molecules path through the polymeric matrix [71]. The addition of OLA plasticizer with carvacrol as active agent on the PLA-PHB (85:15) matrix resulted in a strong plasticizing effect and, thus, in a significant increase in oxygen diffusion rate [67].

One interesting approach to improve the barrier performance of materials is the development of nanocomposites, since nanoparticles are able to create a more tortuous path that increases the effective path length for small molecules, such as gas and vapors [122,123]. In fact, PLA oxygen barrier performance has been improved by the incorporation of CNC as well as functionalized CNC (CNC-s) by the use of a surfactant showing a reductions in OTR*e values of about 43% and 48%, respectively [120]. The same strategy was then use to develop PLA-PHB based nanocomposites loaded with CNC and CNC-s. While PHB produced a reduction of OTR*e value of PLA of about 55%, the addition of CNC or CNCs to PLA-PHB blend did not provoke major changes in PLA OTR*e value [5]. The further addition of ATBC plasticizer to nanocellulose loaded PLA-PHB nanocomposite blends produced an increase of about 80% of the OTR*e value of PLA-PHB-CNCs-ATBC [5] with respect to the un-plasticized nanocomposite PLA-PHB-CNCs [28], but a reduction up to 20% with respect to the OTR*e value of neat PLA [120].

All these findings highlight the advantage of blending PLA with PHB to increase the overall crystallinity in the system. The crystals generate a more tortuous path for small molecules at the same time as the developed crystallites rather enclose the amorphous chains restricting their mobility and reducing the small molecule access [106,124]. Thus, PLA-PHB based formulations result attractive for food packaging purposes were barrier to oxygen is critical to avoid or reduce oxidative processes as well as for low humidity requirements [22].

#### 4.2.5. Migration Properties and Active Packaging Systems

In materials intended for food packaging both the starting substances, including monomers, additive, technological aids, etc., as well as finished plastic material formulations must have regulatory approvals for food contact applications, based on their specific chemical and toxicological features [9]. Regarding legislation applicable to potential migrants from PLA, lactic acid is included in the list of authorized substance with no restrictions or specifications [125]. In fact, lactic acid is present in many foods, both naturally or as a product of in situ microbial fermentation (i.e., yogurt, buttermilk, sourdough breads, and many other fermented foods) [109]. Even so, food packaging materials have to ensure that the total amount of non-volatile substances that might transfer into foodstuff from the polymeric materials does not represent a risk to the consumer [71]. Thus, the legislation establish a maximum limit of 60 mg/kg of food [126]. Overall migration tests were conducted for PLA-PHB (85:15) blends in non-polar and polar food simulants and the obtained overall migration values were well below the legislative limit [71].

Nevertheless, due to the fact that the consumer’s demand for higher quality and longer shelf life food products, there is a continuous raising tendency in the food packaging field to take advantages of the migration phenomenon of active additives with interest in food leading to an increase on research in new active packaging formulations used to improve the quality and safety of food during storage [83,127,128,129,130]. Thus, the release of active substance from the packaging material to the foodstuff could be considered as the exception of the rule, since the transfer of selected additives (such as antioxidants, antimicrobials, aroma compounds, vitamins, etc.) to foodstuff could provide beneficial effects [67].

In this sense, the catechin release from PLA-PHB (75:25) and plasticized with 15 wt % of ATBC (PLA-PHB-ATBC) matrices has been studied into a fatty food simulant. It was observed that the plasticizer increased about three times the release capacity of catechin from the PLA-PHB matrix after 10 contact days at 40 °C. These findings have been ascribed to the ability of plasticizer to increase the polymer chain mobility and, as a result, the release capacity of the polymeric matrix. Finally, the antioxidant effectiveness followed comparative tendency to the catechin release, as demonstrates the antioxidant measurements determined by means of the reduction of the 2,2-diphenyl-1-picrylhydrazyl (DPPH) radical [93]. The DPPH method is one of the most used to determine the antioxidant activity in the active food packaging field mainly because it is simple, inexpensive and robust technique [131]. Additionally, it allows monitoring the inhibition of the radical DPPH oxidation, which can be neutralized either by direct reduction via single electron transfer (SET) or by radical quenching via hydrogen atom transfer (HAT), and in nature SET and HAT mechanisms almost occur together [132].

PLA-PHB (85:75) based matrix has been also used for the development of antibacterial active packaging materials by the incorporation of carvacrol. Plasticized PLA-PHB blends loaded with carvacrol showed their effectiveness against *Staphylococcus aureus* (Gram-positive) and *Escherichia coli* (Gram-negative) as revealed the agar diffusion method as well as by in vitro studies such as the direct contact of materials with both bacterial suspensions for the determination of CFU/mL. However, for un-plasticized PLA-PHB systems no bacterial inhibition was observed by means of the agar diffusion method, but some inhibitory effect against both bacterial strains was observed by in vitro studies [67]. In this sense, the inherent antimicrobial activity of active substance into the polymeric matrix is influenced by morphological, physical and/or chemical characteristics [133]. It has been shown that the antimicrobial sensitivity of *S. aureus* and *E. coli* depends on their chemical interaction with carvacrol exposed into the polymeric matrix surface or released from the polymeric matrix to the foodstuff [127,134]. The agar diffusion method simulates food wrapping, indicating what might happen when the antimicrobial agent migrates from the film to the food product in direct contact with [67]. However, the effectiveness of the polymeric matrices as carriers of antimicrobial compounds does not only depend on the nature of the active agent, but also on the capacity of the film to release sufficient amount of the active compound to the foodstuff at a determined contact time and at equilibrium [134]. In this sense, the presence of plasticizer OLA at 15 wt % significantly enhanced the antibacterial activity of PLA-PHB (85:15) based films due to both, the increased mobility of the macromolecular chains promoting the diffusion of carvacrol and the decrease in the hydrophobic character of the material due to the hydroxyl groups of carvacrol, leading to a higher release of the active agent to the food simulants [67]. Thus, although carvacrol is a volatile compound and high amounts can be lost during melt processing, it has been demonstrated that it remains in its chemical form and could be used for antibacterial functionalities in PLA-PHB blends [70].

### 4.3. Biodegradation of PLA-PHB Polymers Blends

Although the wastes of both biopolymers, PLA and PHB, can be composted, incinerated or recycled [42,135], it seems that PLA-PHB blends are more appropriate for disposal rather than recycling. In this sense, it should be mentioned that consumers have low information about where they have to throw away this kind of plastics after their useful life. Thus, they are commonly disposed with traditional waste plastics. Additionally, biopolymers can result in contamination of traditional recycled plastics, thus interfering with plastic recycling efforts [136]. In this context, one of the main advantages of PLA-PHB blends is that they are able to complete biological degradation in different mediums, such as composting or landfill, which allows reducing the plastic wastes in landfills [7]. The polymers disintegration in compost is managed by aerobic fermentation that ultimately results in humus-rich soil [92]. Meanwhile, the degradation of waste in landfills is mediated by anaerobic fermentation, which is considerably less odorous than its aerobic counterpart, and it produces methane [137]. Although methane could be used as an energy source, it is still not well managed in most current waste management systems.

The biodegradation behavior of PLA and PHB under composting conditions are strongly dependent on the temperature of the composting medium. Actually, while the addition of PHB speed up the biodegradability of PLA at room temperature [40], its delays the PLA biodegradability at 58 °C [92]. This is because PLA and PHB exhibit different degradation mechanisms. PLA degradation is mainly started with a non-enzymatically hydrolysis, which is strongly temperature dependent, that leads in a significantly molar weight reduction followed by the enzymes action throughout the whole of the PLA sample [138]. Indeed, the presence of various enzymes (i.e., proteinase K, serine protease from the fungus Tritirachium album, lipase, esterase and alcalase) can accelerate the PLA degradation [139]. PHB degradation is mainly enzymatically degraded by various enzymes that erodes PHB sample surfaces [40]. In fact, when microorganisms are in contact with PHB secrete enzymes that break the polymeric matrix into successively smaller segments, thereby reducing the average molecular weight [35]. For this reason, the hydrolytic degradation of PHB is accelerated by the presence of PLA component [56].

**Figure 4:**
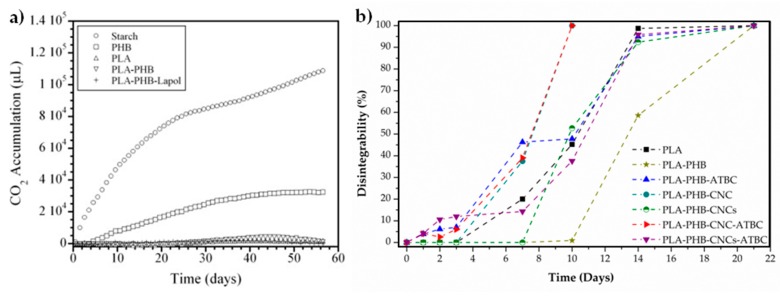
(**a**) Carbon dioxide evolution of the neat PLA, neat PHB, neat Starch (control), PLA-PHB and PLA-PHB-Lapol7% blends as a function of time. Reprinted with permission from [66]; (**b**) Disintegrability degree of neat PLA and PLA-PHB based films under composting conditions as a function of time. Film thickness: 20–30 μm. Adapted from [5,28].

In this context, Zhang et al. followed the degradation of PLA, PHB and PLA-PHB blends samples buried in compost at room temperature. They observed an increment in weight at beginning of the study attributed to water absorption by both biopolymers, which was greater for neat PHB than for neat PLA. The weight change value of PLA resulted almost constant after the first three weeks, indicating that the PLA samples may not biodegrade at room temperature. For neat PHB induction time was required for its biodegradation and a rapid weight loss over a period of eight weeks was observed. In the case of PLA-PHB blends, while for the PLA-PHB 75:25 formulation, a gradual biodegradation occurred from Week 23, PLA-PHB 50:50 and 25:75 formulations showed the induction time period was also observed and it resulted shortly (of about 14 weeks) [40]. Similarly, the evolution of carbon dioxide generated during composting at room temperature was used to follow the biodegradation of neat PLA, neat PHB, PLA-PHB blend and plasticized PLA-PHB with 7 wt % of Lapol 108 using wheat starch as control material (Figure 4a). While neat PLA did not generate much carbon dioxide, neat PHB exhibited an increase in carbon dioxide content up to approximately 40 days, and then this content leveled off thereafter. PLA-PHB blends showed similar behavior than that of PLA, indicating that PLA containing formulations did not degrade much under the experimental conditions [66].

On the other hand, Arrieta et al. followed the mass loss as a measurement of the disintegration degree of PLA-PHB, plasticized PLA-PHB blends [22,92] and their nanocomposites [5,28] under composting conditions at 58 °C at laboratory scale level (Figure 4b) following the ISO standard UNE EN ISO 20200 [140]. The materials disintegrability started in the polymers amorphous phase and this was evidenced by the loss of transparency at the initial stage of the composting process [5,22,92]. The increase of PLA crystallinity due to the PHB presence delayed the PLA degradation rate, since the ordered structure in the crystalline fractions could retain the action of microorganisms [22,92]. Conversely, the plasticizers speeded up the disintegration phenomenon. It seems that the plasticizers, which are mainly lost during the initial disintegration stages, favor the surface hydrolysis leading to substantial losses on the structural and mechanical features, which also made the further disintegration easier [92]. The degradation kinetics of PLA-PHB based polymers is mainly controlled by the surface features such as wettability and roughness during the initial degradation stages, since the disintegrability starts by the PLA hydrolysis process [7]. It will be successively affected by the increased crystallinity due to PHB component [92], since the water diffusion through the polymeric matrix is also highly affected by the overall crystallinity of the polymeric matrix [80]. The addition of hydrophilic nanofillers, such as cellulose nanocrystals, accelerates the degradation process in good relationship with the more hydrophilic character of the nanocompsoites as revealed by the water contact angle measurements [28]. Nevertheless, the better dispersed surfactant functionalized nanocellulose joined with the overall increased crystallinity of the PLA-PHB-CNCs nanocomposite [43] reduced the water diffusion through the polymeric matrix, and, thus, the PLA-PHB-CNCs nanocomposite was disintegrated better than the PLA-PHB-CNC nanocomposite counterpart [28]. In fact, the better dispersed nanocellulose favors the interaction between PLA and PHB matrices by means of hydrogen bonding interactions, exposing lower OH-groups on the material surface which results less polar. Accordingly, the polymeric PLA-PHB matrix becomes less available for water attack during the initial stages of disintegration in compost [5,28]. Similarly, Burgos et al. studied the disintegrability under composting conditions of PLA-PHB (85:15) blends as well as PLA-PHB plasticized with OLA and carvacrol (PLA-PHB-15OLA-10Carv). They observed higher signs of degradation accompanied with higher percentage of disintegration in PLA-PHB-15OLA-10Carv than in PLA-PHB and attributed this behavior to the high chain mobility in plasticized blend provided by OLA and carvacrol [67].

On the other side, PLA has also been blended with amorphous PHB (atactic poly[(R,S)-3-hydroxybutyrate]) and further biodegraded under industrial composting conditions. It was observed that the addition of 15 wt % of amorphous PHB component in PLA samples accelerated the degradation process under composting [57].

## 5. Conclusions

The PLA performance is substantially improved by melt blending it with high crystalline isotactic PHB. The small processing window of PHB represents the foremost drawback for the industrial production of PLA-PHB blends. The PLA-PHB blends processability as well as the thermal stability can be improved by plasticization, adding microparticles and nanoparticles, which finally lead to an improvement of the small processing window of PHB. The use of natural plasticizer is gaining considerably interest in the sustainable food packaging industry. The preparation of preformed masterbatches improves the processability of PLA-PHB based blends. Furthermore, PLA crystallize much faster by blending with PHB in combination with loading fillers (i.e., talc) or nanofillers (i.e., nanocellulose). Although the molecular weight has influence on the miscibility of PLA-PHB, it is less significant than the processing temperature. In this sense, it seems that PLA-PHB blend in 75:25 proportion presents optimal miscibility in the melt state, due to the transesterification reactions.

PHB is able to crystallize PLA leading to materials with higher barrier performance and better mechanical resistance. However, for film manufacturing PLA-PHB plasticization is required and the improvements in the PLA performance caused by semi-crystalline PHB is counteracted by the plasticizer presence. Thus, further strategies such as the development of composites and nanocomposites are frequently used to get packaging materials that have sufficient conditions for processing as well as to obtain materials with a good balance between structural and functional properties able to protect foodstuff during transport and storage. The use of different proportion of PLA and PHB in the blends as well as the addition of plasticizers or filler allows designing formulations with specific performance depending on the requirements of the intended use, as some properties can be modulated by varying the blend composition. PLA-PHB based formulations can also be used as carrier of active compounds (i.e., antioxidant and antimicrobials) for the development of active packaging systems, offering the opportunity to extend a food product’s shelf life.

Thus, formulations based on PLA-PHB blends, including plasticized PLA-PHB systems, composites and nanocomposites, are promising materials that can be prepared through currently used melt processing technologies. Moreover, PLA-PHB is a fully biobased material that also offers composting under aerobic conditions as a sustainable end-life option. The degradation process strongly depends on the composting temperature since PLA degrades faster at 58 °C, while PHB degrades faster at room temperature.

## Figures and Tables

**Table 1:** Tensile test properties of several PLA-PHB based materials.

Formulation	*E* (MPa)	*TS* (MPa)	ε*_B_* (%)	References
PLA	1200–3500	39–42	1.5–8	[61,66,79]
PHB	1670–2600	35–50	2–4	[22,23,61,106]
PLA-PHB 85:15	1220 ± 140	31.0 ± 5.0	100 ± 40	[70,71]
PLA-PHB 75:25	1400–1800	16–50	2–13	[22,23,28,66]
PLA-PHB 50:50	-	8 ± 1	11 ± 2	[40]
PLA-PHB 25:75	-	2.5 ± 1	6 ± 2	[40]
PLA-PHB-CNC (75:25):5	900 ± 50	26.7 ± 2.1	30.0 ± 3.5	[28]
PLA-PHB-CNCs (75:25):5	1900 ± 200	46.5 ± 4.4	80 ± 10	[28]
PLA-PHB-Lapol (75:25):5	1150 ± 40	13 ± 2	15.5 ± 2.0	[66]
PLA-PHB-Lapol (75:25):7	1120 ± 60	15 ± 1	15.1 ± 3.0	[66]
PLA-PHB-Lim (75:25):15	630 ± 20	20.7 ± 1.4	8.0 ± 0.2	[53]
PLA-PHB-PEG (75:25):15	550 ± 25	16.5 ± 3.5	6.0 ± 0.1	[23]
PLA-PHB-ATBC (75:25):15	400 ± 20	14.0 ± 1.8	180 ± 35	[23]
PLA-PHB-ATBC-CNC (75:25):15:5	600 ± 100	27.3 ± 2.9	30.0 ± 3.5	[5]
PLA-PHB-ATBC-CNCs (75:25):15:5	500 ± 20	28.2 ± 8.4	150 ± 15	[5]
PLA-PHB-Carv (85:15):10	1130 ± 60	24.3 ± 1.7	105 ± 25	[70]
PLA-PHB-OLA (85:15):15	1120 ± 60	23.0 ± 2.0	35 ± 14	[71]
PLA-PHB-OLA (85:15):20	950 ± 130	18.0 ± 3.0	220 ± 100	[71]
PLA-PHB-OLA (85:15):30	590 ± 50	19.0 ± 3.0	370 ± 20	[71]
PLA-PHB 70:30	3400	34.6 ± 7.3	12.4 ± 3.3	[61]
PLA-PHB-MA (70:30):1	3345 ± 45	29.5 ± 9.3	31.7 ± 8.6	[61]
PLA-PHB-MA (70:30):3	3327 ± 67	25.5 ± 5.5	48.9 ± 5.7	[61]
PLA-PHB-MA (70:30):5	3015 ± 54	25.4 ± 9.6	365 ± 11	[61]
PLA-PHB-MA (70:30):7	3020 ± 49	22.6 ± 9.3	540 ± 33	[61]
PLA-PHB-MA (70:30):9	3018 ± 71	15.2 ± 4.5	448 ± 47	[61]
PLA-PHB-MA-C30B (70:30):7:1	4107 ± 49	33.5 ± 9.2	503 ± 43	[61]
PLA-PHB-MA-C30B (70:30):7:3	4222 ± 55	43.6 ± 9.2	488 ± 46	[61]
PLA-PHB-MA-C30B (70:30):7:5	3977 ± 98	25.8 ± 8.5	377 ± 41	[61]
PLA-PHB-MA-OMMT (70:30):7:1	4167 ± 85	37.2 ± 3.5	457 ± 66	[61]
PLA-PHB-MA-OMMT (70:30):7:3	4332 ± 43	48.3 ± 5.6	458 ± 12	[61]
PLA-PHB-MA-OMMT (70:30):7:5	3424 ± 23	19.6 ± 6.0	313 ± 23	[61]

**Table 2:** Oxygen barrier performance and Water Vapor Transmission (WVT) values of several PLA-PHB based materials.

Formulation	OTR*e (cm^3^ mm·m^−2^·day^−^^1^)	References	WVT (kg m·s^−^^1^ m^−^^2^ Pa)	References
PLA	30.0–44.5	[79,94,120]	1.3–1.8 × 10^−14^	[67,118]
PHB	11.5 ± 4.5	[22]	7.9–9.5 × 10^−15^	[119,121]
PLA-PHB 85:15	14.9 ± 0.8	[67]	1.5 ± 0.2 × 10^−14^	[67]
PLA-PHB 75:25	24.9 ± 3.8	[22]	-	-
PLA-PHB-CNC (75:25):5	15.3	[28]	-	-
PLA-PHB-CNCs (75:25):5	13.0	[28]	-	-
PLA-PHB-Lim (75:25):15	53.9	[53]	-	-
PLA-PHB-PEG (75:25):15	62.9 ± 1.3	[23]	-	-
PLA-PHB-ATBC (75:25):15	22.8 ± 2.8	[23]	-	-
PLA-PHB-ATBC-CNCs (75:25):15:5	23.3	[5]	-	-
PLA-PHB-Carv (85:15):10	20.7 ± 0.8	[67]	1.4 ± 0.2 × 10^−14^	[67]
PLA-PHB-OLA (85:15):20	25.5 ± 2.1	[71]	1.2 ± 0.1 × 10^−14^	[71]
PLA-PHB-OLA (85:15):30	18.6 ± 1.4	[71]	1.0 ± 0.1 × 10^−14^	[71]
PLA-PHB-OLA-Carv (85:15):15:10	63.3 ± 2.8	[67]	2.0 ± 0.1 × 10^−14^	[67]
PLA-PHB-OLA-Carv (85:15):20:10	76.0 ± 2.7	[67]	1.9 ± 0.3 × 10^−14^	[67]
